# Sprint and upper limbs power field tests for the screening of low bone mineral density in children

**DOI:** 10.3389/fphys.2022.1066462

**Published:** 2022-12-08

**Authors:** Júlio B. Mello, Augusto Pedretti, Gabriel G. Bergmann, Anelise R. Gaya, Esther Ubago-Guisado, Adroaldo C. A. Gaya

**Affiliations:** ^1^ EFiDac Group, Physical Education School, Pontificia Universidad Católica de Valparaíso, Valparaíso, Chile; ^2^ PROESP-Br Group, Universidade Federal do Rio Grande do Sul, Post Graduate Program in Human Movement Science, Porto Alegre, Brazil; ^3^ Laboratório de Estudos Em Esportes Coletivos, Universidade Federal de Pelotas, Pelotas, Brazil; ^4^ Escuela Andaluza de Salud Pública (EASP), Instituto de Investigación Biosanitaria ibs. GRANADA, Granada, Spain; ^5^ Epidemiology and Control of Chronic Diseases, CIBER of Epidemiology and Public Health (CIBERESP), Madrid, Spain

**Keywords:** physical fitness, bones and bone tissue, physical fitness testing, child, bone, classification

## Abstract

**Background:** The possibility of carrying out screening, with acceptable accuracy, of a child’s bone mass status based on a physical fitness test can advance the concept of health-related physical fitness. In addition, the relevance of the applicability of this type of screening in educational environments is mainly due to the difficulty of direct assessments of bone health indicators. This study aimed to propose cut-off points for physical fitness tests based on children’s bone health indicators.

**Methods:** This is a two-phase cross-sectional study. Phase-1: 160 children (6–11 years-old) performed the 20-m sprint test (20-mST) and the 2 kg medicine ball throw test (2 kgMBTT). Areal bone mineral density (aBMD) and content was assessed by DXA. The area under the ROC curve greater than 70% was considered valid. Phase-2: It was carried out a secondary analysis in a sample with 8,750 Brazilians (6–11 years-old). The percentile values (identified in phase-1) were used to identify the values of the cut-off points in the unit of measurement of the tests. The validation of the cut-off points found was by odds ratio values and *p* ≤ 0.05.

**Results:** Phase 1: The areas under the ROC curve were 0.710, 0.712 (boys and girls–20-mST), 0.703, and 0.806 (boys and girls–2 kgMBTT) with total spine and pelvis aBMD as the outcome. Phase 2: From percentile values, we find valid cut-off points in the Brazilian sample (OR > 3.00; *p* < 0.001) for boys and girls. Values ranged between 5.22 s–4.00 s to 20-mST and between 125.0 cm–160.0 cm to 2 kgMBTT. Conclusion. The 20-mST and the 2 kgMBTT presented sufficient accuracy for the screening of children aged between 6 and 11 years with greater chances of having low aBMD in the total spine and pelvis, with valid cut-off points.

## 1 Introduction

Globally, osteoporosis is one of the most relevant health problems, with high morbidity and mortality (Rizzoli et al., 2001). Because its development is related to low bone mass and marked deterioration of bone tissue, it is a consensus that optimizing the accumulation of bone mass during childhood and adolescence, through physical exercise, is an effective way to prevent osteoporosis and maintain adequate bone health in adulthood (Rutherford, 1999). In this way, three recent systematic reviews (Torres-Costoso et al., 2020; García-Hermoso et al., 2021; Mello et al., 2021) indicated the muscular strength, global physical fitness and vigorous physical activities practice as useful skeletal health markers during development and maturation.

Data from a 15-year longitudinal study showed that during adolescence and early adulthood, of all physical fitness variables, only power and isometric strength and speed were related to bone mineral density (BMD) in adulthood (Kemper et al., 2000). As Gómez-Bruton et al. (2017) showed that the increase in lower limb power through plyometric exercises (effects on muscle power) in children and adolescents causes positive effects on bone mineral content (BMC). As a consequence, the WHO 2020 guidelines on physical activity and sedentary behavior indicate for children and adolescents at least three days a week of vigorous-intensity activities that strengthen muscle and bone (in addition to 60 min a day of moderate-vigorous physical activity) (Bull et al., 2020).

In addition to interventions that include physical activity and exercise, national surveys that include measures of health-related physical fitness are an important prevention and monitoring strategy for population health indicators, as indicated in the 2012 American Institute of Medicine report (Committee on Fitness Measures and Health Outcomes in Youth et al., 2012). In this sense, a recent systematic review (Fraser et al., 2021) showed that there are only 13 articles that validated cut-off points for musculoskeletal physical fitness in young peopled. Furthermore, of the articles included in the review, only two (Baptista et al., 2016; Saint-Maurice et al., 2018) used bone health outcomes for cut-off point’s propositions.

In this way, the possibility of carrying out screening, with acceptable accuracy, of a child’s bone mass status based on a physical fitness test can advance the concept of health-related physical fitness. In addition, the relevance of the applicability of this type of screening in educational environments is mainly due to the difficulty of direct assessments of bone health indicators (Shepherd et al., 2011). These assessments are usually carried out in a clinical context by examining bone densitometry by dual-energy x-ray absorptiometry (DXA). In addition to the high cost of this exam, its application in children requires the use of software specifically developed to assess this audience, which in many services becomes an impediment to its applicability (Shepherd et al., 2011). In this sense, to make it possible for professionals working with human movement to have a simple tool for assessing bone mass indicators, this study aims to propose cut-off points for physical fitness tests based on children’s bone health indicators.

## 2 Methods

### 2.1 Study design

This two-phase cross-sectional study with a quantitative approach was carried out after approval by Universidade Federal do Rio Grande do Sul Research Ethics Committee, under opinion 3.414.512 (CAAE-Brazil: 12222019.9.0000.5347, Brazil). In the first phase, field research was carried out with data collection related to the physical fitness and bone health of 160 students. Such information was used for performing the analysis of cut-off points proposition for the physical fitness variables. In the second phase, conducted to identify and validate the cut-off points proposed in phase 1, secondary analyses were carried out in a database consisting of a representative sample of Brazil with 8,750 children.

### 2.2 Phase 1

#### 2.2.1 Research subjects and data collection procedure

In this phase, the convenience sample consisted of 160 children. These are students aged 6–11 years from the 1st to the 5th year of the public elementary school in the city of Porto Alegre, Brazil. The convenience of this sample is justified because the school has a formal partnership with the School of Physical Education, Physiotherapy and Dance at the Universidade Federal do Rio Grande do Sul, where the research was conducted.

To identify the test’s power from the sample size of 160 children, *a posteriori* sample calculation was performed using the G-Power version 3.1 program. For that, we use the equation directed to the proposed association test. The calculation was performed for tests of the F family, considering that the research project foresees association analyses, carried out in another study (Mello, 2020). The alpha used was 0.05, the effect size used was f^2^ = 0.15 (moderate) (Mello et al., 2021), and eight variables as predictors. From this protocol, considering the sample size of the present study, the power of the test (1-β) identified was 0.95.

For data collection, contact was made with the school’s representatives. After signing the authorization terms, all children enrolled from the 1st to the 5th year of elementary school received an invitation to participate in the research and the consent terms. After this stage, a class period was scheduled with each class to carry out the physical tests. The DXA exam was performed at the Exercise Research Laboratory (LAPEX) at the Universidade Federal do Rio Grande do Sul, Brazil. For this purpose, an evaluation time was scheduled with the parents/guardians. This procedure was carried out at the beginning of the academic years of 2017 and 2018.

#### 2.2.2 Test variables

The procedures for collecting physical fitness variables were performed according to the PROESP-Br (Projeto Esporte Brasil) Guidelines for Measurements, Tests and Assessments (Gaya et al., 2021). The physical fitness variables tested were: 1) Sprint, assessed with the running test at a maximum speed of 20 m (20-m sprint test–20-mST); 2) Agility, assessed through the 4 × 4 meter square test (4x4-m square test); 3) Lower Limb Power, assessed using the horizontal jump test, and 4) Upper Limb Power, assessed using the 2 kg medicine ball throw test (2 kgMBTT). These tests also have validation and international use with good evidence (Davis et al., 2008; Bös and Schlenker, 2011; Calleja-González et al., 2015) and are widely used in Brazil (Pedretti et al., 2020).

For the 20-mST, the track was marked with three parallel lines on the ground as follows: starting line, timing line (20 m), and finishing line (2 m after the second line). The third served to avoid the deceleration before the timing line. The appraiser was positioned slightly beside the timing line. The adolescents performed the test twice and the evaluator noted the best time in seconds to two decimal places.

For 4 × 4-m square test, a four-meter square is marked on the side with a cone at each angle. The student started from the standing position. At the signal, the student should move at full speed and touch the cone located on the diagonal of the square. Then, you should run to touch the cone on your left (or right) and then the other diagonal. Finally, it should run towards the last cone. The appraiser was positioned slightly beside the start/finish cone. The adolescents performed the test twice and the evaluator noted the best time in seconds to two decimal places.

For the horizontal jump test, performed with a measuring tape fixed to the ground, perpendicular to a starting line. The adolescents were placed immediately behind the line, with feet parallel, slightly apart, knees semi-flexed, trunk projected in front. At the signal, the adolescents should jump as far as possible, landing with both feet simultaneously. The adolescents performed the test twice and the evaluator noted the best performance in cm to one decimal place.

For 2 kgMBTT, a measuring tape was fixed to the ground perpendicular to the wall. The student sits with knees outstretched, legs joined and back fully supported by the wall. The student holds a medicine ball (2 kg) next to the chest with your elbows bent. At the signal of the evaluator, the student throws the ball as far as possible, keeping his back against the wall. The result is recorded in centimeters to one decimal place.

#### 2.2.3 Outcomes

BMC and areal bone mineral density (aBMD) were collected from the analysis of body composition according to the recommendations of the manufacturer of the DXA device of the GE Healthcare model, Lunar Prodigy (Madison, United States). A trained researcher and a laboratory technician qualified carried out the examinations and in the handling of the device. The device was calibrated once a day before the evaluation sessions. Calibration is performed automatically by the device after positioning the auxiliary calibration cubes. Children were instructed to remove any metal material and wear clothes without zips, buckles or buttons. The evaluator placed the subjects in the supine position and asked them to remain motionless during the measurement, for 5 min, while the equipment arm passed over the body in the head-foot direction.

The values were automatically calculated using the equipment’s software. The values BMC (eg: 70.23 g) and aBMD (eg: 0.978 g/cm^2^) have been described for the total body, total body less head, trunk, total spine, arms, pelvis and legs. These variables were categorized in each body segment was converted to Standard Deviation (SD) values adjusted for sex and age. After that, the variables were classified as “risk for low bone mass” (value ≤ -1 SD) and “normal bone mass” (value > -1 SD). We used the -1SD value because the sample size is not appropriate for using the children’s recommendations (-2SD). This strategy was used by Gracia-Marco et al. (2011) in a study with 380 healthy Spanish adolescents about physical activity and bone mass.

#### 2.2.4 Covariables

Due to the influence that bone mass indicators suffer from biological variables (Heaney et al., 2001), the total body fat percentage (TBF%) and maturity offset were considered covariates. The TBF% was made available during the DXA exam, along with bone variables. For maturity offset calculation, the following variables were required: height, body mass, sitting height, and length of lower limbs. The data collection for these variables and the calculation of maturity offset followed the recommendations proposed by Mirwald et al. (2002).

### 2.3 Phase 2

#### 2.3.1 Research subjects and data collection procedure

The second phase was carried out based on a secondary analysis of a database. This study is part of the “Projeto Esporte Brasil” study (PROESP-Br). The PROESP-Br is a repeated cross-sectional surveillance study that was carried out since 1999. During 2003 and 2009, the Ministry of Sports through the National Secretariat of High-Performance Sports of Brazil funded the PROESP-Br. It was designed to evaluate the anthropometry, sports practice and physical fitness levels of Brazilian children and adolescents using a standardized data collection protocol (Gaya et al., 2021) (the same as phase 1 of this study). Throughout the project (1999–2020), the data collection occurred over the years by previously trained volunteers. For all evaluation, children’s parents/guardians authorized their participation signing the written informed. The Universidade Federal do Rio Grande do Sul originally obtained ethics approval for this project in 2000, under register number: 2008010.

For this study, we selected all children aged 6–11 years with valid data evaluated in the period between January 2011 and December 2020. The period of the last decade was chosen to represent the most current data of the project and suppress possible interference caused by the temporal effect on physical fitness levels (Sandercock and Cohen, 2019; Tomkinson et al., 2019). Following these procedures, the sample consisted of 8,750 Brazilian boys and girls.

### 2.4 Data analysis

#### 2.4.1 Phase 1

A descriptive and exploratory analysis was carried out. In this procedure, the Kolmogorov-Smirnov test was performed and average values, SD, absolute and relative frequency and 95% confidence interval (95% CI) were calculated. Differences between sexes and ages were tested with the two-way ANOVA test adjusting for covariates. For the proposal of the cut-off points for the physical fitness variables, the analysis of Receiver Operating Characteristics (ROC) Curves was used. Firstly, the physical fitness variables were converted into SD values adjusted for sex and age (Gracia-Marco et al., 2011). After this procedure, the bone variables already classified were grouped, thus forming the outcome possibilities that would be tested in the analysis of ROC curves: 1) BMC of the total body less head, 2) aBMD of the total body less head, 3) BMC of the upper limbs and trunk, 4) aBMD of the upper limbs and trunk, 5) BMC of the total spine and pelvis, 6) aBMD of the total spine and pelvis, 7) BMC of the pelvis and lower limbs, 8) aBMD of the pelvis and lower limbs, 9) BMC of the upper limbs, trunk and pelvis, 10) aBMD of the upper limbs, trunk and pelvis, 11) BMC of the total spine, pelvis and lower limbs, and 12) aBMD of the total spine, pelvis and lower limbs.

After these procedures, the area under the ROC curves was calculated between the physical fitness variables (one by one, continuous variable) and the outcomes (one by one, categorical variable) stratified by sex and adjusted for maturity offset and body fat percentage. The outcome that presented the largest area under the ROC curve was chosen as the reference for bone health indicator. The analyses for the identification of the cut-off points were performed taking into account that the lower limit of the 95% CI was not less than 0.50. Acceptable values of area under the ROC curve were also considered to be greater than 70% and with significant *p*-values (*p* ≤ 0.05). The cut-off points were defined based on the best balance between sensitivity and specificity. This balance was defined as the greatest sum between sensitivity and specificity, as long as the cut-off points were more sensitive than specific. Thus, percentage values were identified for boys and girls that represent greater chances of low bone mass based on the results of physical tests.

#### 2.4.2 Phase 2

The percentile values (identified in phase 1) were used to identify the values of the cut-off points in the unit of measurement of the tests (e.g., 20-mST, cut-off point in seconds). For this, a descriptive analysis was carried out with the PROESP-Br database and the values corresponding to the percentiles were described for each sex and each age, these being the suggested cut-off points. To verify the validation of the cut-off points found, odds ratio (OR) calculations were used based on the binary logistic regression equation. In this analysis, the outcome variable was the bone health indicator that gave rise to the cut-off point. The independent variable was the physical fitness variable tested, already classified with the proposed cut-off point. The cut-off points that showed an indication of association were considered valid, verified through the 95% CI, the statistical probability value and the OR. Statistical programs IBM^®^ SPSS 20.0 and Stata 13.0^®^ (College Station, TX, United States) were used for all analyses.

## 3 Results

### 3.1 Phase 1

The sample of 160 children had an equivalent distribution between sexes and ages, in addition to having a normal distribution in all variables. Table 1 describes all anthropometric variables, physical fitness, fat percentage, maturity offset and age. The mean values of age, height and weight were similar (*p* > 0.05) by sex. However, maturity offset, TBF% and physical fitness variables showed differences (*p* < 0.05) between boys and girls. These results indicated that there is no need for the ROC curve analysis to be stratified by age, but only by sex.

**TABLE 1:** Description of physical fitness variables, bone outcomes and covariates of the sample used in phase 1 of the study (*n*: 160).

	Total		Boys (85)		Girls (75)	
	X ± SD		X ± SD		X ± SD	
Age (years)	8.9 ± 1.56		8.25 ± 1.65		8.35 ± 1.50	
Height (m)	1.33 ± 0.10		1.34 ± 0.10		1.33 ± 0.11	
Weight (kg)	33.03 ± 10.14		32.80 ± 9.31		33.29 ± 11.03	
Maturity-offset*	−3.02 ± 1.73		−4.01 ± 1.11		−1.95 ± 1.65	
TBF%*	32.66 ± 8.53		30.47 ± 8.61		35.15 ± 7.77	
Sprint (s)*	4.54 ± 0.85		4.45 ± 0.96		4.63 ± 0.70	
Agility (s)*	7.91 ± 1.00		7.70 ± 1.01		8.13 ± 0.94	
Lower limb power (cm)*	110.58 ± 24.04		117.09 ± 24.57		103.54 ± 21.48	
Upper limb power (cm)*	184.23 ± 53.85		191.92 ± 51.83		175.90 ± 55.10	


	**n**	**%**	**n**	**%**	n	%
BMC TBLH						
Normal bone mass	135	85.4	77	91.6	59	78.7
Risk for low bone mass	23	14.6	7	8.4	16	21.3
aBMD TBLH						
Normal bone mass	137	86.7	77	92.8	60	80.0
Risk for low bone mass	21	13.3	6	7.2	15	20.0
BMC upper limbs and trunk						
Normal bone mass	134	84.3	74	88.1	60	80.0
Risk for low bone mass	21	15.7	10	11.9	15	20.0
aBMD upper limbs and trunk						
Normal bone mass	124	78.0	67	79.8	57	76.0
Risk for low bone mass	35	22.0	17	20.2	18	24.0
BMC total spine and pelvis						
Normal bone mass	130	81.3	73	85.9	57	76.0
Risk for low bone mass	30	18.8	12	14.1	18	24.0
aBMD total spine and pelvis						
Normal bone mass	128	80.0	68	80.0	60	80.0
Risk for low bone mass	32	20.0	17	20.0	15	20.0
BMC pelvis and lower limbs						
Normal bone mass	130	81.3	73	85.9	57	76.0
Risk for low bone mass	30	18.8	12	14.1	18	24.0
aBMD pelvis and lower limbs						
Normal bone mass	124	77.5	67	78.8	57	76.0
Risk for low bone mass	36	22.5	18	21.2	18	24.0
BMC upper limbs, trunk and pelvis						
Normal bone mass	129	81.1	72	85.7	57	76.0
Risk for low bone mass	30	18.9	12	14.3	18	24.0
aBMD upper limbs, trunk and pelvis						
Normal bone mass	117	73.6	61	72.6	56	74.7
Risk for low bone mass	42	26.4	23	27.4	19	25.3
BMC total spine, pelvis e lower limbs						
Normal bone mass	126	78.8	70	82.4	56	74.7
Risk for low bone mass	34	21.3	15	17.6	19	25.3
aBMD total spine, pelvis e lower limbs						
Normal bone mass	121	75.6	66	77.6	55	73.3
Risk for low bone mass	39	24.4	19	22.4	20	26.7

*Denote a significant difference (*p* < 0.05). *n*: absolute value; X ± SD: average value and standard deviation; %: percentage value; TBF%: total body fat percentage; BMI: body mass index; BMC: bone mineral content; aBMD: areal bone mineral density; TBLH: total body less head.

Regarding BMC and aBMD in each analyzed body segment, even with adjustment, no differences (*p* > 0.05) between the sexes were identified (Supplementary Table S1). The percentage of low bone mass in the total sample varied between approximately 13% and 26% (Table 1). In the most of the outcomes, girls had a higher percentage of low bone mass when compared to boys in about 10 percentage points. There were between 5 and 8 cases of sample loss in the described variables.

**FIGURE 1:**
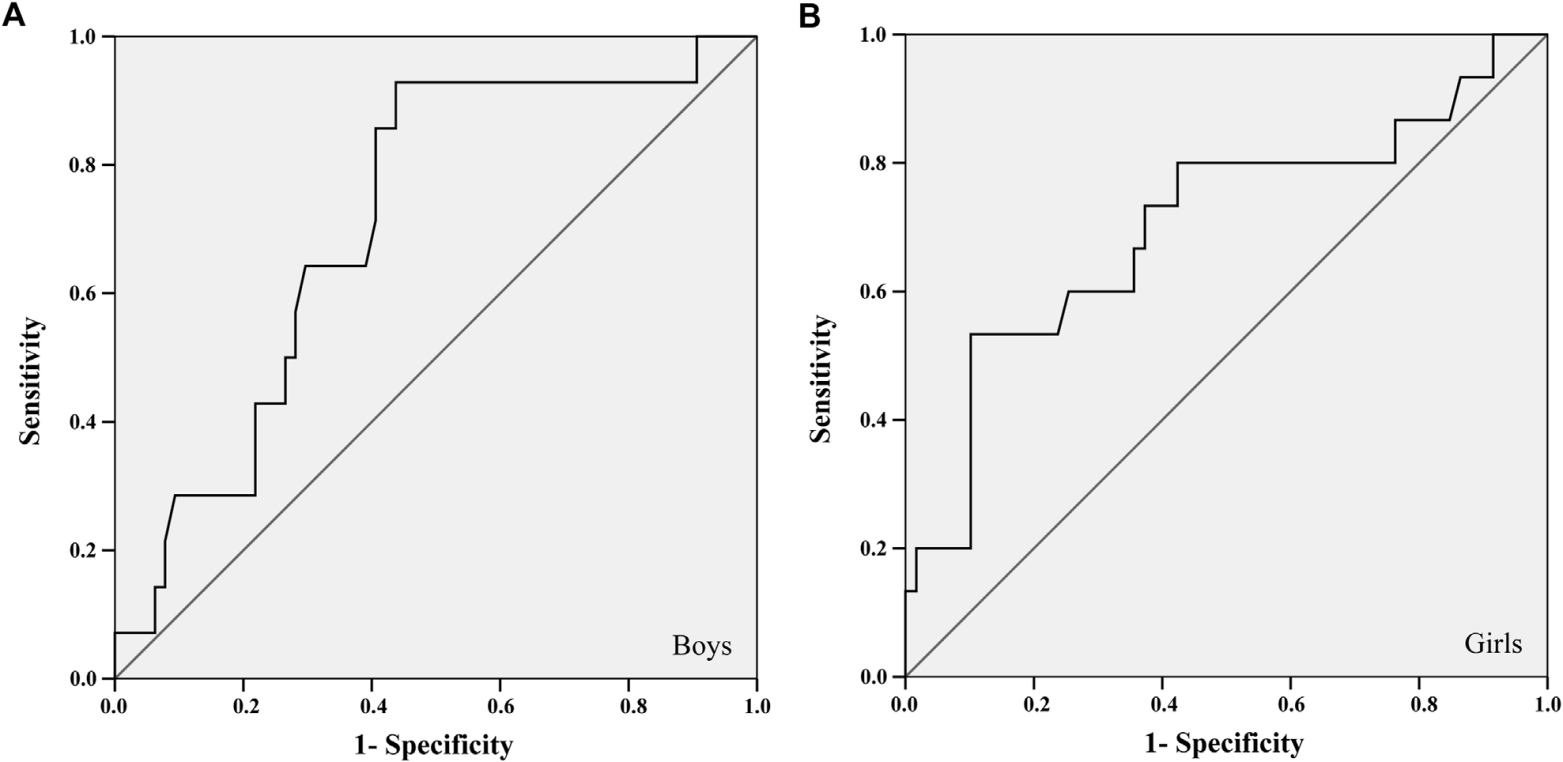
ROC curve between the 20-m sprint test and the areal bone mineral density of the total spine and pelvis of boys **(A)** and girls **(B)**.

**FIGURE 2:**
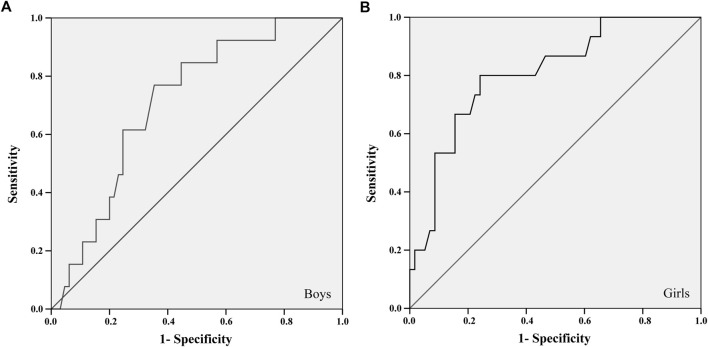
ROC curve between the 2 kg medicine ball throw test and the areal bone mineral density of the total spine and pelvis of boys **(A)** and girls **(B)**.

For the ROC curve analysis, the physical fitness variables were converted into units of SD (Z score) adjusted for age and stratified by sex. Thus, it was possible to position the points of each child on the probability distribution curve without disregarding some differences identified between ages and sexes (Supplementary Tables S2, S3). The largest areas under the ROC curves in all tests were found about the aBMD of the total spine and pelvis, for both sexes. Two tests met all the criteria: 20-mST and 2 kgMBTT (Figures 1, 2).

The 20-mST and 2 kgMBTT showed an area under the ROC curve greater than 0.700 with significant statistical probability value and acceptable confidence intervals for identifying cut-off points (Table 2). The better adjustment point between sensitivity and specificity was identified in the cut-off list provided by the ROC curve analysis (Table 2). For the 2 kgMBTT, the cut-off points for both sexes were those with the highest sum between sensitivity and specificity. In the 20-mST, the cut-off point for boys was the highest sum. For girls, we choose the fourth-highest sum, because the previous three values were more specific than sensitive. From these cut-off values, the percentile corresponding to the standardized value of the tests (Z score) in each sex was identified in the sample.

**TABLE 2:** Synthesis of areas under the ROC curves between the physical fitness variables and the areal bone mineral density outcome of the total spine and pelvis.

Test variable	Area under ROC curve	*p*-value		95% CI
		Boys
20-m sprint	0.710	0.014		0.572–0.848
4 × 4 m square	0.664	0.056		0.504–0.824
Horizontal jump	0.652	0.076		0.513–0.790
2 kg medicine ball throw	0.712	0.016		0.579–0.846
		Girls
20-m sprint	0.703	0.016		0.538–0.867
4 × 4 m square	0.641	0.093		0.477–0.806
Horizontal jump	0.539	0.643		0.385–0.693
2 kg medicine ball throw	0.806	0.000		0.686–0.927
	Cut-off point (Z score)	Sensibility	Specificity	Percentile value
20-m Sprint test				
Boys	−0.173	0.929	0.563	47.5
Girls	−0.038	0.800	0.576	50.0
2 kg medicine ball throw test				
Boys	−0.208	0.769	0.646	42.4
Girls	−0.233	0.800	0.759	35.8

Bold *p*-values denote a significant difference (*p* < 0.05); *p*-value: statistical probability value; 95% CI: 95% confidence interval.

### 3.2 Phase 2

From the secondary analysis of the PROESP-Br database, the values that represent the cut-off points (in the test measurement unit) equivalent to the percentiles described are shown in Table 3.

**TABLE 3:** Bone health-related cut-off points for the 20-m sprint tests and 2 kg medicine ball throw test for children 6–11 years old.

	2 kg medicine ball throw test (centimetres)		20-m sprint test (seconds)	
Age	Boys	Girls	Boys	Girls
6	147.0	125.0	4.81	5.22
7	168.7	140.0	4.52	4.88
8	190.0	158.1	4.31	4.66
9	210.0	175.0	4.25	4.58
10	232.0	202.0	4.09	4.44
11	260.0	228.0	4.00	4.36

In the validation analysis (Table 4), it was possible to see that children classified as the Risk for low bone mass for upper limb power and sprint are about four times more likely to have a low aBMD in the total spine and pelvis than their no-classified peers. When stratified by sex, associations remain with high odds ratios, ranging from 2.88 to 6.63 times more likely to have a low aBMD in the total spine and pelvis.

**TABLE 4:** Association of the speed and upper limbs power with the areal bone mineral density of the total spine and pelvis of children.

	OR	95% CI	*p*-value
Total sample			
Sprint			
Health zone	1	–	–
Risk for low bone mass	4.25	1.80–10.0	0.001
Upper limb power			
Health zone	1	–	–
Risk for low bone mass	3.95	1.46–7.86	0.004
Boys			
SPRINT (s)			
Health zone	1	–	–
Risk for low bone mass	4.26	1.26–14.40	0.020
Upper limb power			
Health zone	1	–	–
Risk for low bone mass	3.75	1.062–13.23	0.040
Girls			
SPRINT (s)			
Health zone	1	–	–
Risk for low bone mass	4.21	1.26–14.04	0.019
Upper limb power			
Health zone	1	–	–
Risk for low bone mass	3.59	1.10–11.68	0.034

Bold *p*-values denote a significant difference (*p* < 0.05); OR: odds ratio; 95% CI: 95% of confidence interval.

## 4 Discussion

The main evidence from this study indicates that for children aged 6–11 years, the 2 kgMBTT and 20-mST were accurate enough to identify cut-off points for screening children with greater chances of having a low aBMD in the total spine and pelvis. From these results, it was possible to establish valid cut-off points for these tests from a secondary analysis of a national database. Thus, we emphasize that the main practical application of this study is the proposition of cut-off points for physical fitness tests that can be applied in the school environment, training, pediatric care, or any place that deals with the child’s health (Fraser et al., 2021). The use of physical fitness tests for the initial screening of the bone health profile of children is a simple strategy, cheap and does not require the use of sophisticated equipment in primary healthcare. Such information may help Physical Education teachers, for example, to carry out screening in the school environment, since the assessment of bone mineral density has a high cost. Therefore, greater care could be devoted to children who are not in the healthy test zone.

### 4.1 Physical fitness and bone health

Some studies have shown that the level of physical activity is associated with bone development variables (Tan et al., 2014; Gómez-Bruton et al., 2017; García-Hermoso et al., 2021), in addition, some studies specifically address the relationship between physical fitness and bone health indicators in childhood. Current evidence (Torres-Costoso et al., 2020) supports that muscular strength is a marker of skeletal health in children and adolescents, besides, available evidence from longitudinal studies demonstrates that only muscle strength and speed in childhood are related to BMD in adulthood (Kemper et al., 2000; Barnekow-Bergkvist et al., 2006).

Our study proposed an association, based on ROC curve analyses, between physical fitness and bone health indicators. The organization of outcomes took into account the potential regional effect of physical exercise on bones (McKay et al., 2000; Fuchs et al., 2001; Vicente-Rodriguez et al., 2004; Lu et al., 2009). However, it is noteworthy that literature data on the association between physical fitness and bone mass are still controversial. Although longitudinal studies have pointed out specific physical capacities as influencing factors in long-term osteogenesis (Kemper, 2000; Vicente-Rodríguez et al., 2008; Khawaja et al., 2019), other studies have shown that only muscle fitness (Sayers et al., 2011; Torres-Costoso et al., 2020) is associated with BMD, although others have found a relationship with cardiorespiratory fitness (Dib et al., 2005; Ginty et al., 2005; Vicente-Rodríguez et al., 2008).

The associations between bone mass and physical fitness variables can be based on evidence on the osteogenic effect in childhood of some types of physical exercise (Rutherford, 1999; Vicente-Rodríguez, 2006). Different controlled trials in school and non-school settings demonstrate that although the intensity is important, the type of activity also has an important influence on the osteogenic effect (Tan et al., 2014; Larsen et al., 2018; García-Hermoso et al., 2021; Mello et al., 2021). This influence takes place through the actions of the reaction force of the different activities.

### 4.2 Physical fitness and screening aBMD

Some studies suggest that the greater the ground reaction force in activities, the greater the osteogenic stimulus (Rutherford, 1999; Kontulainen et al., 2002; Gómez-Bruton et al., 2017). Thus, if children perform jumping activities, the lower limbs and lumbar spine tend to have a greater osteogenic effect, unlike ball receptions, punches and grips, which would suffer the action of reaction forces from other bodies.

Even so, it is necessary to be cautious when interpreting associations, taking into account the physical fitness test that was performed and what it might represent in the child’s usual activities (Bull et al., 2020; Gómez-Bruton et al., 2020). In this sense, our results indicated that the 20-mST and 2 kgMBTT had acceptable accuracy for screening children with increased chances of low aBMD of total spine and pelvis. There is a strong point in using these two tests because together they represent different reaction force possibilities–ground or not–by the lower and upper limbs.

The 20-mST assesses the child’s speed, but it also represents the ability to high-intensity run, which tends to reflect their usual activities. High-intensity runs are multiple jumps with a specific pattern of ground reaction force, which can lead the test to represent an osteogenic effect that has already occurred in children for up to months (Janz et al., 2003; Sutter et al., 2019). Similarly, the 2 kgMBTT assesses the power of the child’s upper limbs (Davis et al., 2008). Furthermore, it represents the muscle’s ability to produce force (Harris et al., 2011), which can occur naturally (hormonal production) or through stimuli for activities that require strength (adaptation through the neural pathway in the case of prepubertal children). It is also assumed that the result of this test may be a reflection of the child’s usual activities.

Both tests mentioned have adequate validity criteria for children (Davis et al., 2008; Calleja-González et al., 2015), in addition to being described in national (Pedretti et al., 2020) and international studies (Davis et al., 2008; Harris et al., 2011). Even so, the proposition of cut-off points for muscle speed and power tests based on a BMD variable is in line with the results reported by longitudinal studies discussed above (Kemper et al., 2000; Barnekow-Bergkvist et al., 2006). In agreement with our results, current evidence (Gómez-Bruton et al., 2020) demonstrated that upper and lower limb muscle strength, in addition to speed/agility predicted all measured bone variables, except for volumetric BMD. The authors strongly suggest that global fitness is an essential determinant of bone structure and strength in preschool children. Furthermore, they indicate that physical fitness testing could provide useful information related to bone health in children.

### 4.3 Limitations and strengths

Our results, despite being innovative, must be analysed based on the knowledge of the study’s limitations. The children’s level of physical activity and sports practice was not controlled, so the explanations for the relationship between physical fitness and bone outcomes may be due to other interferences. As all the children were in the same maturity offset group (they did not reach the growth peak), even with an adjustment for maturation, the analysis is still fragile and future studies must include children at different maturational levels in the sample. The use of -1SD value for the first classification of bone health (phase 1) is a good strategy for a small sample size but is not a clinical recommendation. A future study with a larger number of children and considering vitamin-D intake may be able to better explore the tangent relationships with physical capacities that were not related to this study. Finally, it is natural that the results of cross-sectional studies be analysed with caution, as it is not possible to attribute a cause-and-effect relationship.

Despite the aforementioned limitations, it is important to consider that this study has strengths that should be highlighted. The results described show applicability in different contexts related to primary care for children. Another important fact is that the gold standard assessment of body composition was used to assess the main outcomes, showing the reliability of the results. Finally, phase 2 of this study, where the cutoff points were identified and validated, was carried out with a database of more than 8,000 children from all regions of Brazil, adding characteristics of representativeness to the sample and the results found.

## 5 Conclusion

The 20 mST and the 2 kgMBTT presented sufficient accuracy for the screening of children aged between 6 and 11 years with greater chances of having low aBMD in the total spine and pelvis. From these physical fitness tests, it was possible to identify valid cut-off points for boys and girls. In this way, these results have important practical applicability, in which we suggest the use of tests and cut-off points in all primary healthcare settings, schools, schools and sports clubs, in addition to pediatric clinics.

## Data Availability

The datasets presented in this study can be found in online repositories. The names of the repository/repositories and accession number(s) can be found below: proesp.ufrgs.br.
